# Supplementary material for: Content-rich biological network constructed by mining PubMed abstracts
Source: BMC Bioinformatics. 2004 Oct 8;5:147. doi: 10.1186/1471-2105-5-147 (PMC528731; doi:10.1186/1471-2105-5-147)
Supplement: Additional File 2 — The original results of the above study (non-essential files are deleted to keep the file size under the limit set by BMC bioinformatics). [file 1471-2105-5-147-S2.bz2 › chilibotAdditionalFile2/dip10/34ID7566124E118/html/CASP3_GZMB.html]

 


 **CASP3** and **GZMB** 
  
Found 75 abstracts in PubMed, retrieved 10.  
 

 What does Google say? 
 PDF only 
| .edu only 

---

**Interactive relationship** (e.g. stimulation, inhibition, etc)

**Inhibitory relationship**- Thus granzyme B  [ **GZMB** ]  mediates direct cleavage of caspase 3  [ **CASP3** ]  and also activates mitochondrial disruption, resulting in the release of proapoptotic proteins that suppress caspase inhibition.  Ref: 12648453 Immunity, 2003
**Neutral relationship**- Once in the cytosol, granzyme B  [ **GZMB** ]  targets caspase 3  [ **CASP3** ]  directly or indirectly through the mitochondria, initiating the caspase cascade to DNA fragmentation and apoptosis.  Ref: 12752668 Immunol Rev, 2003
- Recent work shows that granzyme B  [ **GZMB** ]  mediated release of apoptotic factors from the mitochondria is essential for the full activation of caspase 3  [ **CASP3** ] .  Ref: 12752668 Immunol Rev, 2003

**Non-interactive relationship** (e.g. studied together, co-existance, homology, etc.)

- Actual execution of apoptosis depends on proper functioning of effector caspases, particularly caspase 3  [ **CASP3** ] , and on the expression levels of apoptosis regulating proteins, including Bcl 2 and the recently identified granzyme B  [ **GZMB** ]  specific protease inhibitor 9 PI9.  Ref: 12036886 Blood, 2002
- The DS brain was examined using immunocytochemistry and antibodies against the active fragment of caspase 8 AC, 8 and to caspase 3  [ **CASP3** ]  cleavage products of fodrin CCP  [ **GZMB** ] , a neuronal cytoskeleton protein.  Ref: 12213643 Neurosci Lett, 2002
- Treatment of HL 60 cells with lactacystin, a selective inhibitor of the proteasome, exponentially increased caspase 3  [ **CASP3** ]  like hydrolytic activity and induced apoptosis but had little or no effect on the activity of upstream caspase 8, caspase 9, or granzyme B  [ **GZMB** ] .  Ref: 12869638 Mol Pharmacol, 2003
